# Metabolites and gene expression in the myocardium of fasting rats in an acute hypoxic environment

**DOI:** 10.1186/s12864-023-09309-1

**Published:** 2023-05-10

**Authors:** Ruzhou Zhao, Xiaobo Wang, Xiang Zhou, Shuai Jiang, Lin Zhang, Zhibin Yu

**Affiliations:** 1 Beijing Institute of Biotechnology, Academy of Military Medical Sciences (AMMS), Beijing, China; 2 Department of Aerospace Physiology, Air Force Medical University, Xi’an, China; 3 Department of Nuclear Medicine, Xijing Hospital, Air Force Medical University, Xi’an, China

**Keywords:** High altitudes, Fasting, Myocardium, Extreme hypoxia, L-glutamine

## Abstract

With the rising demand for entry to extremely high altitudes (HAs), rapid adaptability to extremely hypoxic environments is a challenge that we need to explore. Fasting was used to evaluate acute hypoxia tolerance at HA and was proven to be an effective method for improving the survival rate at extreme HA. Our experiments also showed that fasting pretreatment for 72 h significantly increased the 24 h survival rate of rats at 7620 m from 10 to 85% and protected the myocardium cells of rats. Here, we compared the metabolites and gene expression in the myocardium of SD rats pretreated with fasting and nonfasting at normal altitude and extreme HA. Our findings demonstrated that the dynamic contents of detected differential metabolites (DMs) between different rat groups were consistent with the expression of differentially expressed genes (DEGs), and DM clusters also showed strong correlations with DEG clusters. DM clusters related to amino acids and lipids were significantly lower in the fasting groups, and the correlated DEG clusters were enriched in mitotic pathways, including *CDK1*, *CDC7*, *NUF2*, and *MCM6*, suggesting that fasting can attenuate mitotic processes in cardiac tissues and reduce the synthesis of amino acids and lipids. L-Glutamine-related metabolites were particularly low at extreme HA without pretreatment but were normal in the fasting groups. The DEGs in the cluster related to L-glutamine-related metabolites were enriched for T-cell receptor V(D)J recombination, the Hippo signaling pathway, the Wnt signaling pathway, the cGMP-PKG signaling pathway, and the mTOR signaling pathway and were significantly downregulated, indicating that the content of L-glutamine decreased at extreme HA, while fasting increased it to adapt to the environment. Moreover, abundant fatty acids were detected when rats were exposed to extreme HA without pretreatment. Our study revealed the fasting and hypoxic environment-related factors in SD rats and provided new insights into the genetic and molecular characteristics in the myocardium, which is critical to developing more potential rapid adaptation methods to extreme HA.

## Introduction

High altitudes (HAs) are generally considered to be altitudes higher than 1500 m above sea level, with the main feature of hypobaric hypoxia [1]. HA is further classified as high (1500–3000 m), very high (3500–5500 m), and extremely high (5500–8850 m) [2]. High-altitude sickness usually occurs during a rapid ascent above 2500 m, characterized by impaired physical performance and increased ventilation frequency. Acute mountain sickness (AMS) is the most common high-altitude sickness and is usually accompanied by anorexia, nausea, dizziness, malaise, sleep disturbance, or a combination of these symptoms [3]. With the increase in altitude, the risk of severe high-altitude illnesses, such as high-altitude cerebral edema (HACE) and high-altitude pulmonary edema (HAPE), are increasing, which are potentially serious diseases for fit individuals [4, 5].

Previous studies have mainly focused on long-term adaptation at Has, while few have focused on rapid adaptation to Has. A period of acclimatization, such as stepped ascent, is considered a useful method but is too time-consuming and not suitable for rapid entry [6]. The progressive deterioration of physiological functions at extreme Has cannot be prevented by adequate training in exercise and rest [2]. In recent years, the need to ascend to extreme HAs and even HAs higher than 7620 m has grown, primarily for work and tourism purposes [7]. Some mechanistic studies on adaptation to hypobaric hypoxia have also been reported [8, 9]. The protein translation process is downregulated under hypoxia, and unnecessary protein synthesis is inhibited to prevent the accumulation of stress-induced unfolded proteins [8]; for example, the mammalian target of rapamycin (mTOR) is involved in the inhibition of protein synthesis [10]. In addition, mitochondrial quality control occurs in response to hypobaric hypoxia [9]. Until now, most adaptations to HA have been focused on entry to very HAs and are not suitable for entry to extreme HAs, such as 7620 m.

At an altitude of 7620 m, the ambient partial pressure of oxygen is 60 mmHg, and the oxygen saturation of human arterial blood drops to approximately 55%, which leads to loss of consciousness due to the alveolar partial pressure of oxygen decreasing to below 30 mmHg after a few minutes [11, 12]. A human cannot stay at this altitude for a long time without protection and medical assistance. The signaling pathways in response to low-pressure hypoxic stimuli in SD rats are highly similar to those in humans. Therefore, the use of the SD rat model to study hypoxic habituation or rapid adaptation to hypoxia can provide a valid experimental basis for human application. Our previous study identified fasting as an effective method to rapidly adapt to hypoxic environments and downregulate mTOR, which is a pivotal factor that contributes to the strengthened adaptability of SD rats to hypoxia [7].

Although fasting significantly improves extreme hypoxic tolerance in SD rats, the duration of fasting limits its practical application in humans. Understanding its mechanisms could help us to develop rapid adaptation to extreme HA. In this study, we used SD rats as a model and simulated the hypobaric hypoxic environment. The metabolites in different treatment SD groups were identified and quantified, and the gene expression was also analyzed by the RNA sequencing method. Additionally, the results of the metabolism and gene expression regulation analyses were combined to reveal the dynamics among different groups and determine the effects of fasting and environment-related factors.

## Materials and methods

### Experimental animals

A total of 115 adult male SD rats (10 − 12 weeks old, body weight > 300 g) were used in this study. Before the experiment, all rats were raised under the same conditions in specific pathogen-free (SPF) cages with suitable temperatures (25 ± 5 °C) and humidity (50 ± 5%). First, to investigate the 24 h survival rate of SD rats at extreme HA, 40 SD rats were equally divided into two groups, nonfasting and fasting pretreatment groups, and were exposed to 7620 m for 24 h. Then, 75 SD rats were assigned to four groups, including Con (rats at baseline altitude, 412 m), F (rats fasted for 72 h at baseline altitude, 412 m), H (rats exposed to a simulated altitude of 7620 m for up to 24 h, until death), and FH (rats fasted for 72 h and then exposed to a simulated altitude of 7620 m for 24 h). To obtain live rats for the echocardiography test and fresh tissue samples, the Con, F, and FH groups were each randomly assigned five rats, while 60 rats were included in the H group based on the low survival rate of the H group in the previous test. Fresh heart and liver tissues were collected from four rats (as replicates) for each group and frozen at − 80 °C (CWBIO, Beijing, China).

The fasting rats were deprived of standard rodent feed but were provided water. The 7620 m altitude was simulated by our laboratory-developed hypobaric chamber system [7], which consists of a control panel, a vacuum pump, a pressure and flow control system, and hypobaric chambers. The chamber temperature and humidity were 25 ± 5 °C and 50 ± 5%, respectively. The pressure altitude was set with the control panel, and then hypobaric hypoxia was mimicked via a vacuum pump. The flow control system controlled the ascending and descending speeds at a rate of 10 m/s. An airflow of 1 L/min per rat was also sustained to ensure fresh air in the chambers. This study was approved by the Ethics Committee for Animal Care and Use of Air Force Medical University.

### Echocardiography

Cardiac function was assessed by M-mode echocardiography using the preclinical small animal ultrasound imaging system (Vinno Corp., Suzhou, China). For the rats without fasting, echocardiography was immediately conducted before ascending (Con) and after descending (H); for the fasting rats, echocardiography was immediately performed before fasting (Con) and after fasting before ascending (F) and after descending (FH).

Before the ultrasonography examination, rats were anesthetized with 3% isoflurane. The heart rate (HR), left ventricular internal diameter at end-systole (LVIDs), left ventricular internal diameter at end-diastole (LVIDd), left ventricular end-diastolic volume (LVEDV), and end-systolic volume (LVESV) were recorded. Left ventricle ejection fraction (LVEF) and left ventricle fractional shortening (LVFS) were calculated as follows: LVEF% = [(LVEDV − LVESV)/(LVEDV) × 100] and LVFS% = [(LVIDd − LVIDs)/(LVIDd) × 100].

### Transmission electron microscopy (TEM)

Fresh cardiac and hepatic tissues were collected from rats, immediately fixed with 4% glutaraldehyde (Servicebio, Wuhan, China) and cut into 0.1 × 0.1 × 0.1 cm^3^ blocks for further fixation with 1% osmium tetroxide in deionized water. Electron micrographs were collected using TEM (HITACHI 7800, Tokyo, Japan) at 80 kV. The morphology of myocardial fibers and mitochondria was observed at 14 × , 35 × , and 84 × magnifications.

### Identification and quantification of metabolites

We identified and quantified metabolites in the myocardium of four groups using a Triple TOF-6600 mass spectrometer and LC20 ultra-performance liquid chromatography. The correlation coefficient between different samples in the same group was larger than 0.9, so the average metabolite levels of all samples was used as the metabolite level of each group in the following analysis. OPLS-DA was performed to detect differential metabolites (DMs) between two groups based on the quantification results, with the criteria of VIP ≥ 1, log2FC ≥ 1, and *p* value < 0.05. The quantitative DMs were subjected to log2 (FPKM + 0.01) transformation and then normalized by z score. The normalized z score values were used to perform hierarchical clustering (HCL), principal component analysis (PCA), and K-means clustering analysis for these DMs.

### RNA library and sequencing

Total RNA was isolated using TRIzol based on the manufacturer’s protocol, and then cardiac samples were treated with DNase to degrade any genomic DNA contaminating the samples. The RNA purity was assessed by 1.2% agarose gel electrophoresis using the RNA 6000 Pico LabChip Kit on the Agilent 2100 BioAnalyzer. The RNA sequencing library was constructed using the NEBNext Ultra II RNA Library Prep Kit. To obtain high-quality libraries, the size distribution of libraries was analyzed using an Agilent 2100 Bioanalyzer, and the quantities of libraries were evaluated using an ABI StepOnePlus Real-Time PCR System. Finally, all RNA libraries were sequenced on the Illumina HiSeq platform with the pair-end module.

### Gene expression analysis

To obtain clean reads, raw sequencing reads were filtered by removing low-quality reads with adapters, more than 20% bases with qualities lower than 10, and unknown bases of more than 10%. To remove the reads of rRNA, the filtered reads were mapped to the Rfam database [13], and the mapping reads were removed. Clean sequencing reads were mapped to the rat gene set, and the fragments per kilobase of transcript per million base pairs sequenced (FPKM) were then calculated for each gene using Rockhopper [14]. The correlation coefficient between different samples in the same group was larger than 0.9, so the average gene expression levels of all samples was used as the gene expression level of each group in the following analysis. Differentially expressed gene (DEG) analysis between any two groups was performed using DEseq2, and DEGs were filtered with fold change ≥ 2.00, probability ≥ 0.8, and p adjust ≤ 0.05.

The gene ontology and pathway functional enrichment analyses of DEGs were performed using the Stats R package [15]. The false discovery rate (FDR) of each *P* value was calculated, and the terms with FDR ≤ 0.01 were defined as significantly enriched. The expression levels of genes were transformed by log2 (FPKM + 0.01) and further processed by zero-mean normalization. Then, hierarchical clustering (HCL), principal component analysis (PCA), and K-means clustering analysis were performed based on the normalized values. Finally, weighted gene coexpression network analysis (WGCNA) was performed for each module, and the genes most related to the DMs were screened [16]. Taking the correlation ≥ 0.9 as the threshold, the correspondence between DMs and DEG clusters was obtained. Ten differentially expressed genes and factors were selected randomly from different metabolic pathways to perform qRT‒PCR validation. Primer Premier 5.0 software (PREMIER Biosoft International, CA, USA) was used to design the qRT‒PCR primers. The housekeeping gene β-actin was selected as an internal control. PCR products were identified by 1.2% agarose gel electrophoresis.

## Results

### Higher survival rate and less myocardial damage in fasting rats at 7620 m altitude

**Fig. 1:**
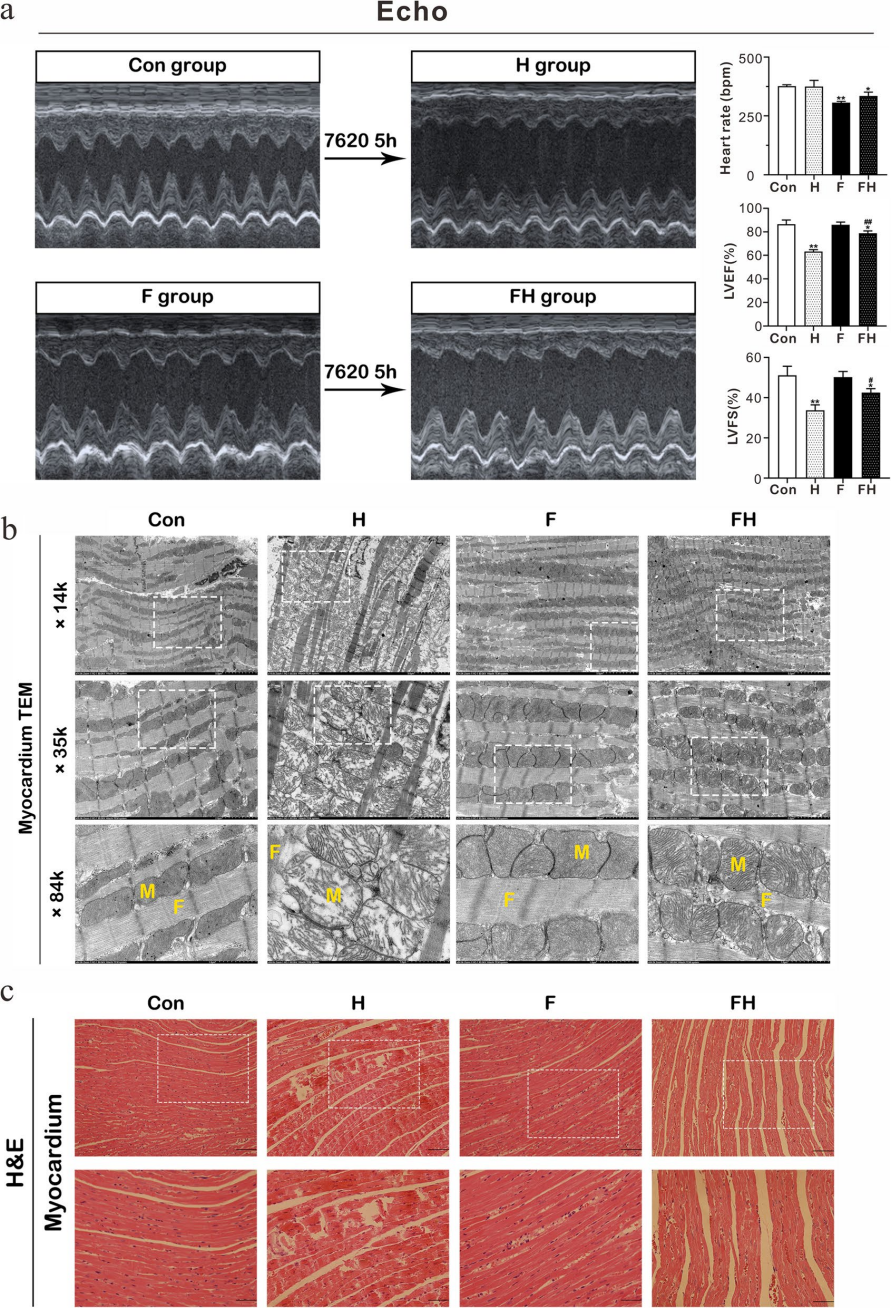
Fasting pretreatment alleviated tissue injuries and maintained cardiac function during acute extreme hypoxia. **a** Myocardial M-mode echocardiography images. SD rats were subjected to echocardiography in the following stages: before hypoxia treatment and after exposure to 7620 m for 5 h (Con-CH groups); before 72 h of fasting, after 72 h of fasting, namely, before hypoxia treatment and after exposure to 7620 m for 5 h (Con-F-FH groups). **b** Electron micrograph showing myocardial fibers and mitochondrial shape. The white-boxed regions are enlarged in the next row. M represents mitochondria, and F represents myocardial fibers in the × 84 K row. Scale bars = 5 μm (× 14 k), 2 μm (× 35 k), and 1 μm (× 84 k). **c** Histological staining (hematoxylin and eosin) of myocardial and hepatic tissue, scale bars = 200 μm and 50 μm (enlarged diagram). The results are representative of 5 rats/group

Similar to a previous study [7], we used SD rats as an in vivo model and simulated the hypobaric hypoxic environment in the present study. Compared to the Con group, the rat survival rate of the F group was significantly improved at 7620 m altitude (Table 1). Cardiac function was assessed by echocardiography. After exposure to 7620 m for 5 h, the LVEF and LVFS were significantly decreased in H and HF groups (Fig. 1a). The abrogated reductions in LVEF and LVFS in the FH group indicated that fasting pretreatment prevented cardiac function impairment caused by acute extreme hypoxia. Electron micrographs of the myocardium showed that the myocardium of the H group had fractured myocardial fibers, severe disorganization, and massively swollen mitochondria with ruptured mitochondrial membranes and disrupted cristae **(**Fig. 1b). Compared to the Con group, the myocardium of the FH group showed slight injury, and the cardiac tissue of the H group was badly damaged, including ruptured cardiomyocytes and disordered myocardial fibers (Fig. 1c). The hepatic tissue of the H group was also impaired, exhibiting a disruption in hepatic lobule structure and irregular liver cell morphology. However, these impaired features were alleviated with fasting pretreatment in the FH group.

**Table 1:** Survival rates of adult SD rats (10 ~ 12 weeks old) after exposure to 7620 m for 24 h

	T	S	R (%)
Con	20	2	10
F	20	17	85

Con, normal SD rats; F, SD rats with fasting pretreatment for 72 h; T, total number of SD rats in experiments; S, survival number of SD rats after exposure to 7620 m for 24 h; R, survival rate of SD rats after exposure to 7620 m for 24 h

### The dynamic content of differential metabolites (DMs) was consistent with the expression of differentially expressed genes (DEGs)

Myocardial damage was significantly less severe in the fasting pretreatment rats than in the rats without fasting pretreatment at 7620 m altitude. Our previous study also found that fasting reduced the production of histones and fat in the myocardium. To detect the dynamics of myocardial metabolism in rats, we identified and quantified metabolites in the myocardium of the four groups and performed DM analysis between all of the groups.

**Fig. 2:**
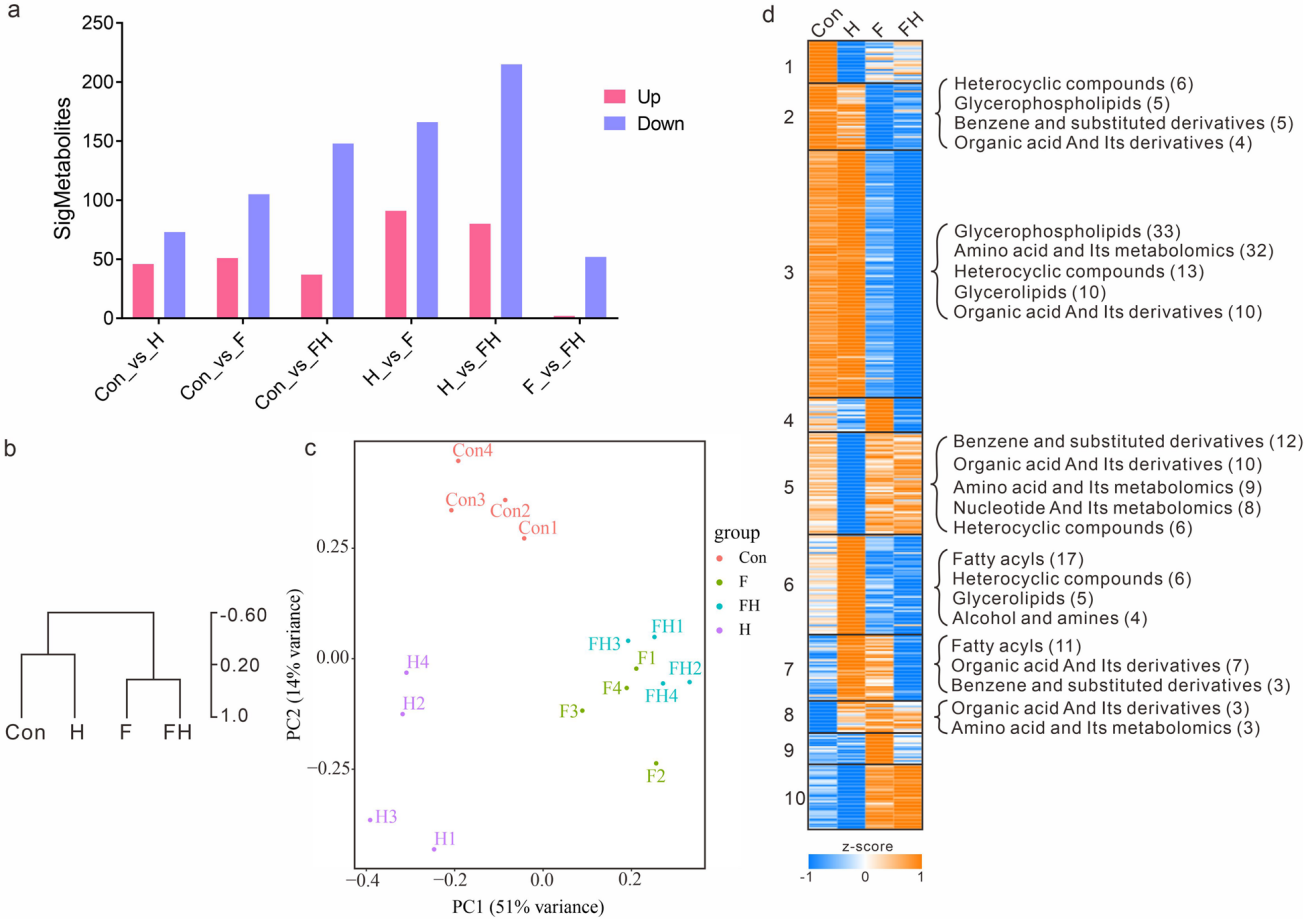
Clustering of DMs. **a** The statistics of DMs between for all two-group comparisons. The red column represents the upregulated DMs, and the blue column represents downregulated DMs. **b** HCL clustering of DMs. **c** PCA results of DMs. The F and FH groups clustered closely. **d** K-means clustering of DMs. A total of ten clusters were determined. The main DMs are labeled at the right for some clusters

A total of 3,191 metabolites were identified in the myocardium in all groups, which were mainly composed of lipids and amino acids, and 447 DMs were screened, mostly including fatty acyls, amino acids, organic acids, and glycerophospholipids. Compared to the Con group, the number of DMs in the H, F, and FH groups were similar (Fig. 2a). More DMs were identified in the H-vs.-F group and H-vs.-FH group comparisons, and the F-vs.-FH group comparison had the fewest DMs. The metabolic patterns were significantly altered in the H and F groups, while the FH group had the same metabolic pattern as that of the F group. To detect the dynamic patterns of DMs, hierarchical clustering (HCL), principal component analysis (PCA), and K-means clustering analysis were performed on 447 DMs. The HCL and PCA results showed that the dynamic patterns of DMs were more similar between the Con and H groups and between the F and FH groups (Fig. 2b, c). A total of ten modules were clustered by K-means clustering, and approximately half of them, including modules 2 and 3, had higher DMs in the Con and H groups and lower DMs in the F and FH groups. Some DMs were higher in the F and FH groups and lower in the Con and H groups, such as those of module 10. Some DMs were the lowest in the H group, such as those of module 5, while some DMs were the highest in the H group, such as those of module 6 (Fig. 2d).

**Fig. 3:**
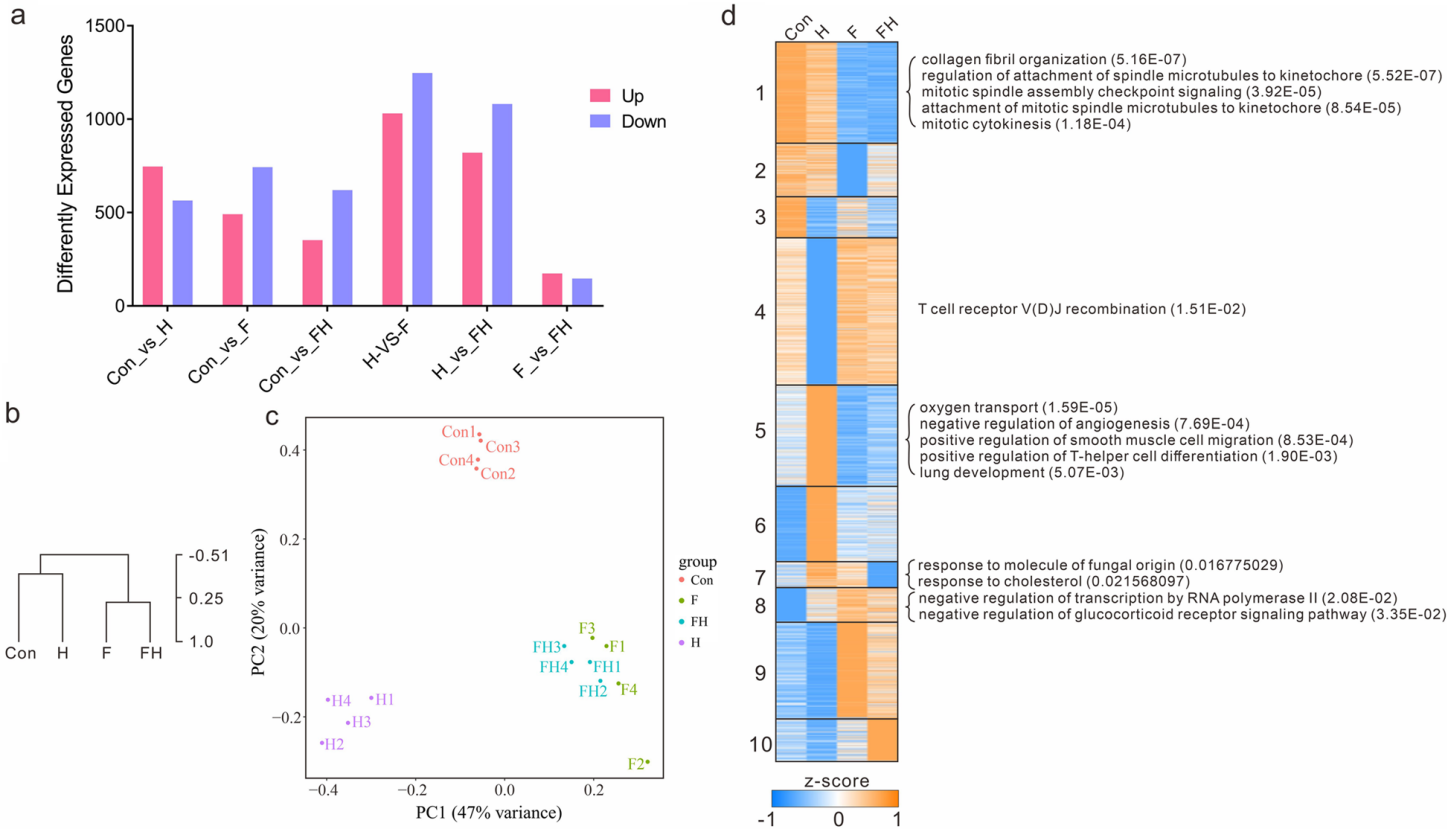
Clustering of DEGs. **a** The statistics of DEGs for all two-group comparisons. The red column represents the upregulated DEGs, and the blue column represents downregulated DEGs. **b** HCL clustering of DEGs. **c** PCA results of DEGs. The F and FH groups clustered closely. **d** K-means clustering of DEGs. A total of ten clusters were determined. The main DEGs are labeled at the right for some clusters

To detect gene expression in the four groups, RNA sequencing was performed for the rat myocardium of each group, and 773.4 M clean reads were obtained for all samples. A total of 19,570 expressed genes were detected, of which the FPKM of 12,470 genes was larger than 1 in at least one group. Finally, 2,832 DEGs were detected for all two-group comparisons. The distribution of DEGs was similar to that of the DMs. Compared to the Con group, the number of detected DEGs in the H, F, and FH groups was similar. The detected DEGs in the H-vs.-F group and H-vs.-FH group comparisons were the most abundant, while the DEGs in the F-vs.-FH group comparison were the least abundant (Fig. 3a). Hierarchical clustering (HCL), principal component analysis (PCA), and K-means clustering analysis were performed based on all detected DEGs (Fig. 3b-d). Ten modules were clustered by K-means clustering and were enriched in the GO and KEGG databases. The clustering results showed that the expression pattern of DEGs was consistent with the dynamic pattern of DMs. The expression patterns of DEGs in the Con-vs.-H group comparison and that in the F-vs.-FH group comparison were similar. The gene expression patterns and the corresponding metabolic content in clustered modules were consistent. According to the gene expression results, the clustered modules were divided into three major classes: Class I, modules highly expressed in the Con and H groups, such as module 1; Class II, modules with differential expression specific to the H group, such as low level expression for module 4 only in the H group and high level expression for modules 5 and 6 only in the H group; Class III, modules highly expressed in the F and FH groups, such as modules 9 and 10.

### Correlation between differential metabolites and DEGs

**Fig. 4:**
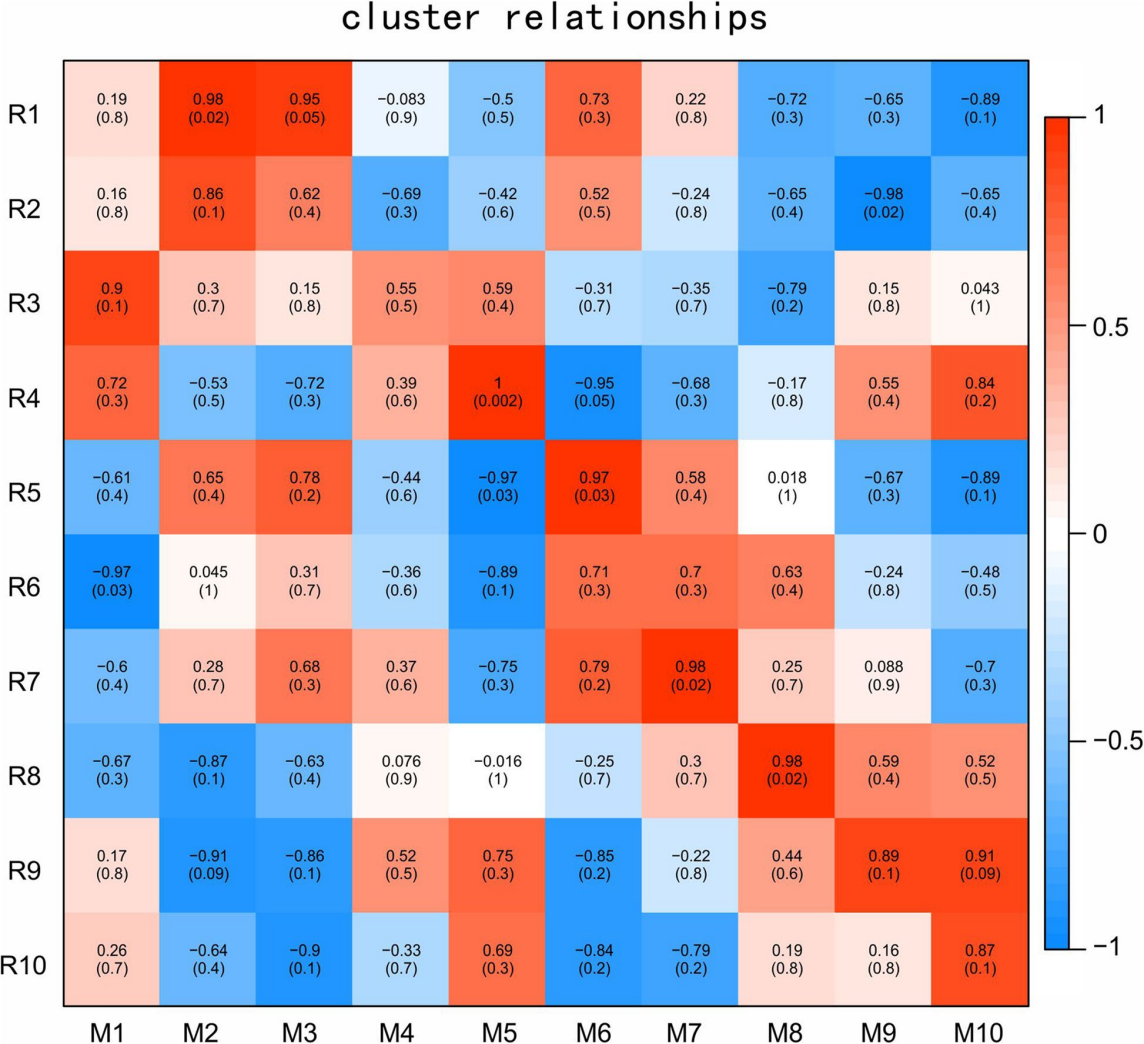
Correlation of K-means clustering modules between DEG clusters and DM clusters. The colors represent the correlation coefficients. From red to blue, the correlation gradually decreases

The dynamic pattern of DMs was consistent with the expression pattern of DEGs. To screen the DEGs most associated with DMs, WGCNA correlation analysis was performed. DEG Cluster 1 was the most correlated with DM Clusters 2 and 3, and DEG Cluster 4 was the most correlated with DM Cluster 5. DEG Cluster 5 was the most correlated with DM Cluster 6, DEG Cluster 7 was the most correlated with DM Cluster 7, and DEG Cluster 8 was the most correlated with DM Cluster 8 (Fig. 4).

### Fasting attenuated mitotic processes in cardiac tissues and reduced the synthesis of amino acids and lipids

**Fig. 5:**
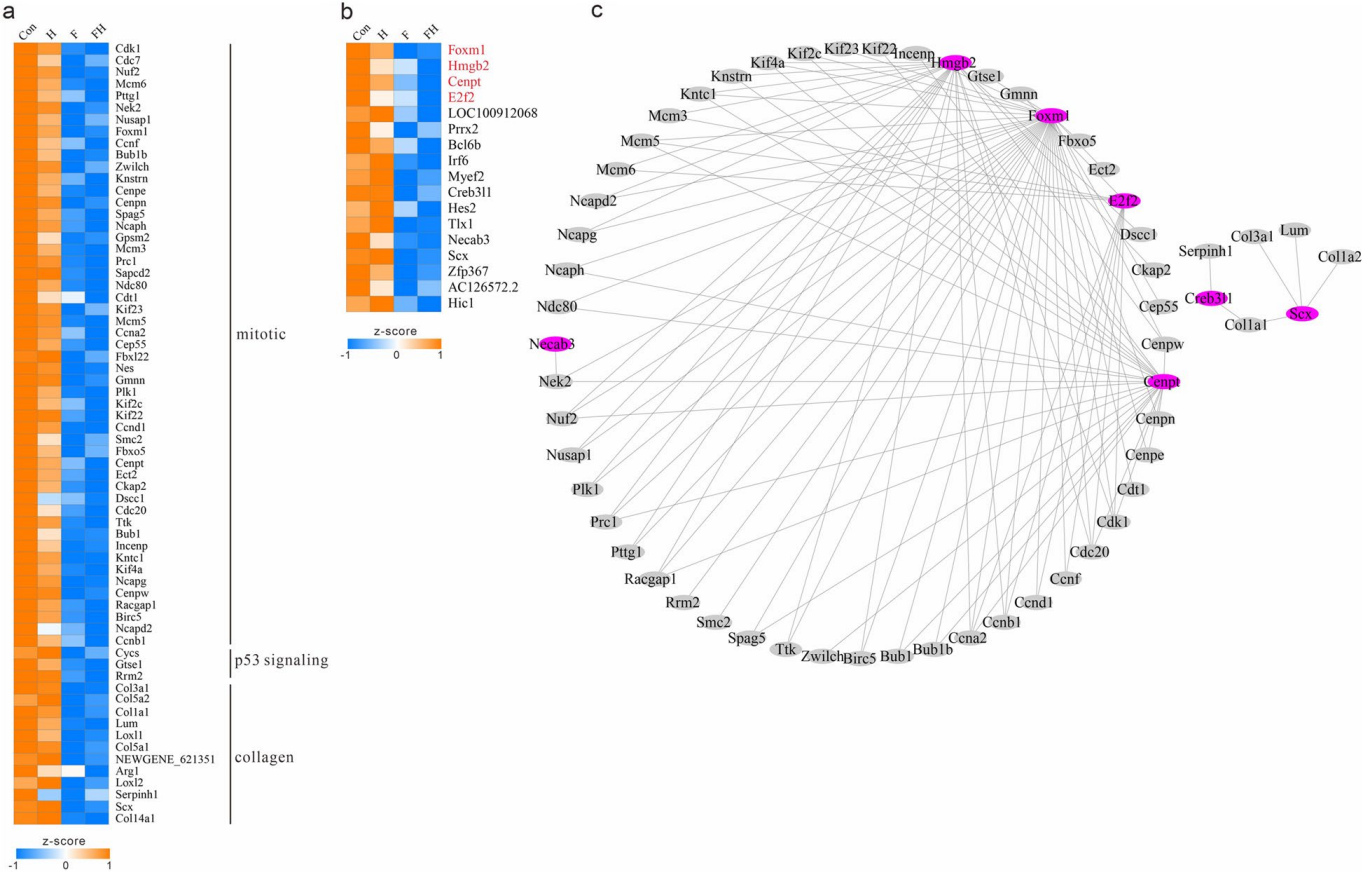
Heatmap of downregulated genes and transcription factors in the fasting groups. **a** Heatmap of downregulated genes in the fasting groups. **b** Heatmap of downregulated transcription factors in the fasting group. **c** PPI interactions between downregulated genes and transcription factors in the fasting group

Among the DM clusters, the DM contents of Clusters 2 and 3 were significantly lower in the fasting groups, and the involved metabolites were mainly glycerophospholipids and amino acids. The main glycerophospholipids were lysophosphatidylethanolamine and lysophophatidylcholine. The main amino acids were Leu, Glu, Lys, Arg, and Phe. DEG Cluster 1 was the most associated with DM Clusters 2 and 3. Genes of DEG Cluster 1 were significantly enriched in multiple mitotic-related pathways, such as regulation of attachment of spindle microtubules to kinetochores and mitotic spindle assembly checkpoint signaling, including 51 associated genes, such as *CDK1*, *CDC7*, *NUF2*, *MCM6*, and *PTTG1* (Fig. 5a). In addition, genes of DEG Cluster 1 were also significantly enriched in the p53 signaling pathway, and the related genes were *CDK1*, *CCND1*, *CCNB1*, *CYSS*, *GTSE1*, and *RRM2*. Some genes of DEG Cluster 1 were also significantly enriched in collagen fibril organization and collagen biosynthesis, and the associated genes were *COL3A1*, *COL5A2*, *COL1A1*, and 12 others. There were 17 transcription factors clustered in DEG Cluster 1, including *IRF6* of the IRF family and *CREB3I1* of the bZIP family. PPI analysis identified four transcription factors, *FOXM1*, *HMGB2*, *E2F2*, and *CENPT,* that have multiple interactions with the identified p53 signaling and collagen pathway-related genes mentioned above (Fig. 5b, c).

### 7620 m altitude decreased L-glutamine content in the myocardium, while fasting increased it

**Fig. 6:**
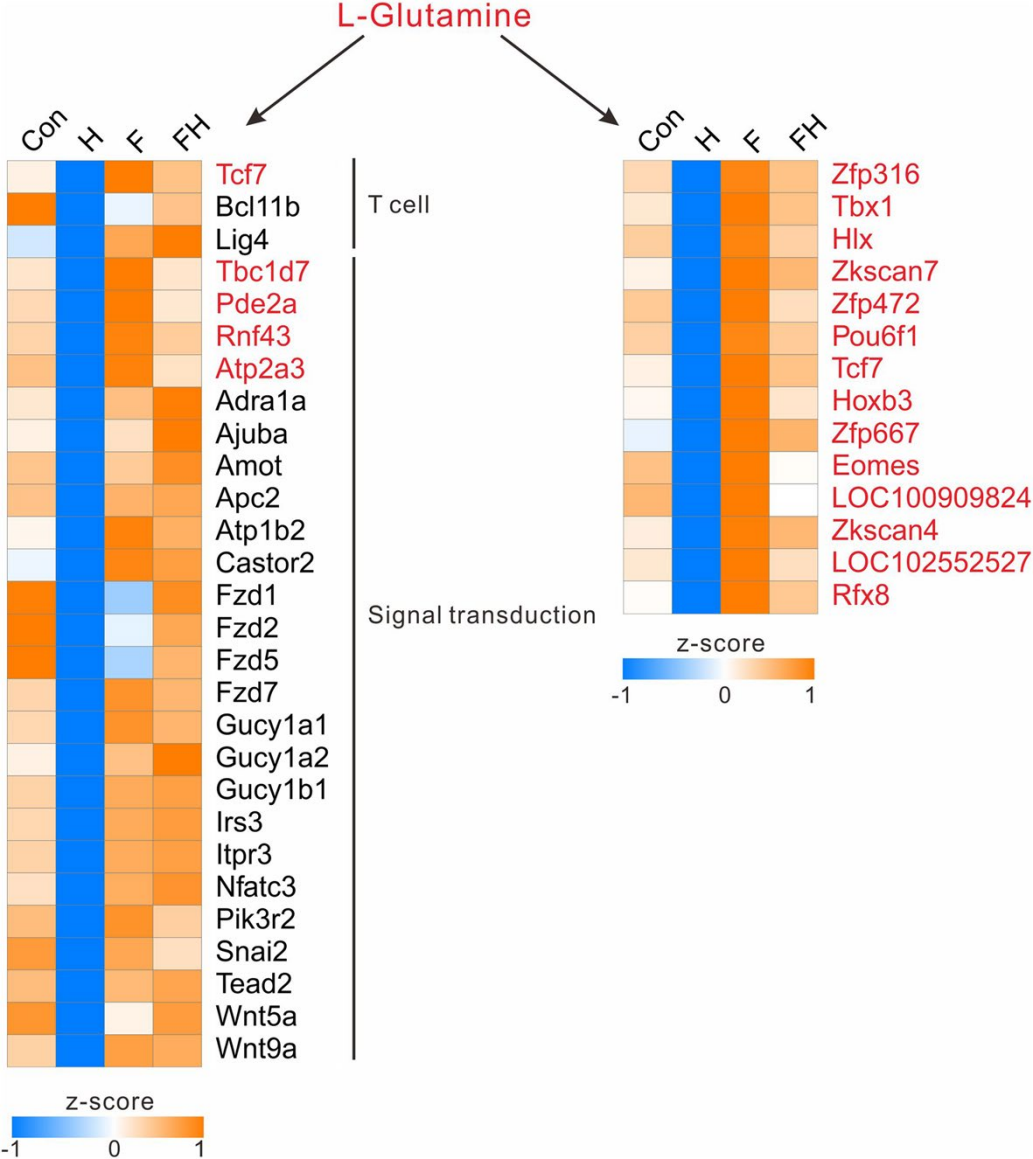
Heatmap of genes and transcription factors most associated with L-glutamine among different groups. The left panel is the heatmap of the most highly related genes, and the right panel is the heatmap of the most highly associated transcription factors

Among the ten DM clusters, the contents of metabolites in Cluster 5 were the lowest in the H group and higher in the other groups. These metabolites were enriched in both the biosynthesis of amino acids and the GABAergic synapse in the nervous system. According to the enrichment results, we found that L-glutamine had particularly importance. Metabolites in Cluster 5 were almost all enriched in the pathway that contained L-glutamine. The contents of L-glutamine were similar in the Con and FH groups but lower in the H group and higher in the F group. Among the ten DEG clusters, Cluster 4 was the most related to DM Cluster 5. Genes in DEG Cluster 4 were also only expressed at low levels in the H group and were significantly enriched in T-cell receptor V(D)J recombination and multiple signal transduction pathways, such as the Hippo signaling pathway, Wnt signaling pathway, cGMP-PKG signaling pathway, and mTOR signaling pathway. In addition, DEG Cluster 4 contained 91 transcription factors. To determine the genes and factors most associated with L-glutamine, WGCNA was performed based on the amount of L-glutamine in different subgroups and the expression of special functional genes and transcription factors. The WGCNA results showed that the genes most associated with L-glutamine were *TBC1D7*, *PDE2A*, *RNF43*, and *ATP2A3*, in which *TBC1D7* was involved in the mTOR signaling pathway, *PDE2A* and *ATP2A3* were involved in the cGMP-PKG signaling pathway, and RNF43 was involved in the Wnt signaling pathway. Fourteen transcription factors were most closely related to L-glutamine, including *HOCB3*, *LOC102552527*, *RfFX8*, *EOMES*, *TCF7*, *ZFP472*, *TBX1*, *LOC100909824*, *ZFP667*, *ZKSCAN7*, *HLX*, *ZKSCAN4*, *POU6F1*, and *ZFP316*, in which *TCF7* was also directly involved in T-cell receptor V(D)J recombination, the Wnt signaling pathway, and the Hippo signaling pathway (Fig. 6). All results indicated that the contents of L-glutamine increased from the Con group to the F group and decreased from the F group to the H group; furthermore, the contents in the FH group reached similar levels to those in the Con group. L-Glutamine is closely related to various signal transduction pathways and transcription factors in the myocardium.

### Fatty acids abundantly accumulated in the H group

**Fig. 7:**
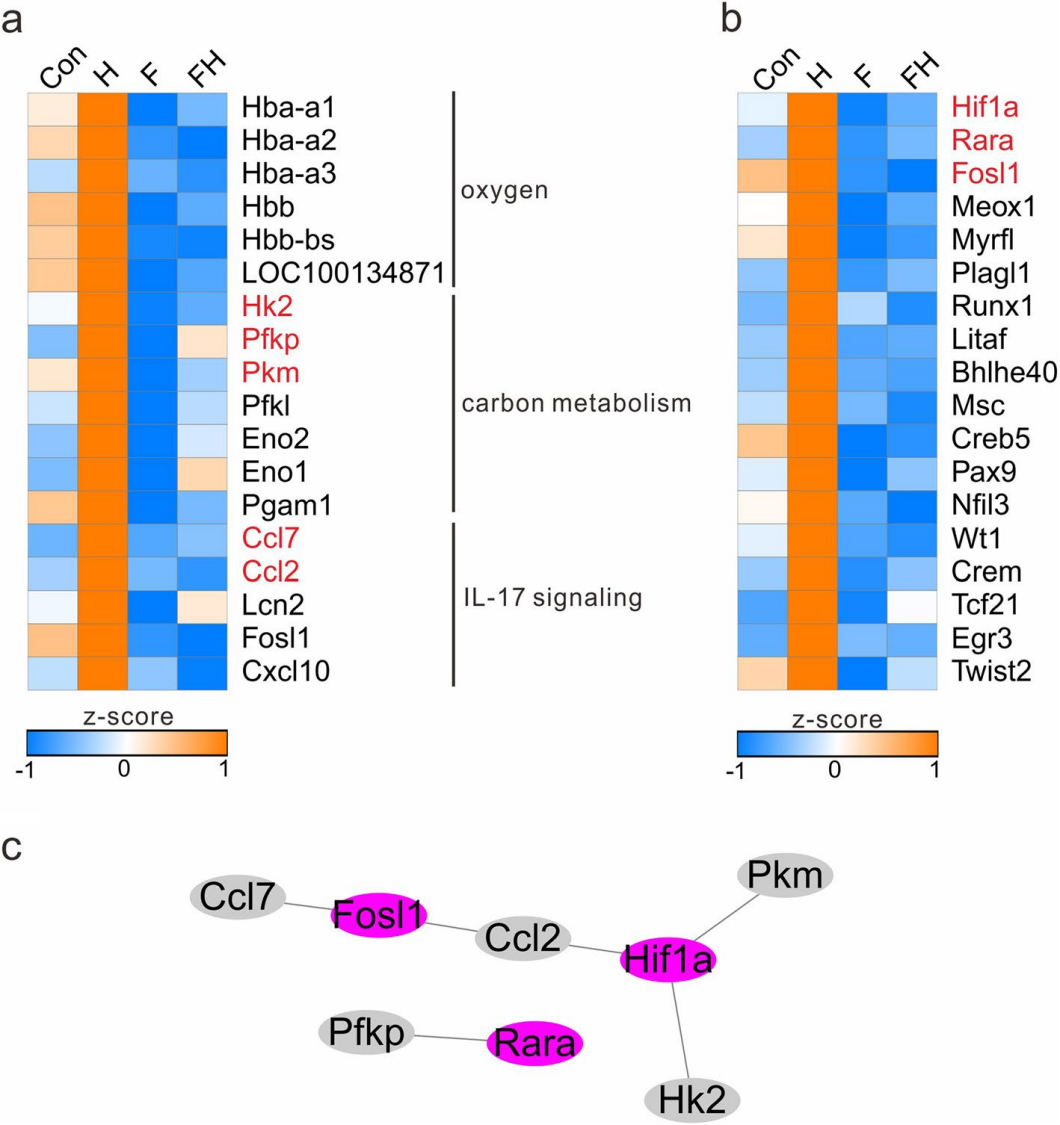
Heatmap of the expression of specific genes and transcription factors highly expressed in the H group. **a** Heatmap of the expression of specific genes. **b** Heatmap of the expression of transcription factors. **c** PPI interactions of specific genes and transcription factors in the H group

The metabolites in DM Cluster 6 were the highest in the H group and lower in the F and Con groups, and these metabolites mainly consisted of free fatty acids and oxidized lipids, which were significantly enriched in several lipid metabolism pathways, such as biosynthesis of unsaturated fatty acids (EPA, DPA, and adrenic acid), fatty acid biosynthesis (EPA), fatty acid biosynthesis (DPA), fatty acid biosynthesis (DPA and adrenic acid), fatty acid biosynthesis (palmitoleic acid and myristic acid), and linoleic acid metabolism (13(S)-HPODE and (7S,8S)-DiHODE). DEG Cluster 5 was the most related to DM Cluster 6, and genes in DEG Cluster 5 were also highly expressed in the H group and were significantly enriched in oxygen-related pathways, such as oxygen transport, oxygen carrier activity, and oxygen binding. Some genes were also significantly enriched in carbon metabolism (metabolism from glucose to pyruvate) and the IL-17 signaling pathway (Fig. 7a). There were 18 transcription factors contained in DEG Cluster 5, and the PPI results showed that *HIF1A* and *RARA* interacted with *HK2*, *PKM*, and *PFKP, which are involved* in carbon metabolism, and *FOSL1* and *HIF1A* interacted with *CCL2* and *CCL7, which are involved* in the IL-17 signaling pathway, but none of the 18 transcription factors interacted with oxygen-related genes (Fig. 7b, c). These analyses showed that oxygen-related genes were activated when rats were placed directly at 7620 m altitude without fasting, the transformation from glucose to pyruvate was enhanced, and then the transformation from pyruvate to fatty acids was also enhanced. In addition, the IL-17 signaling pathway was activated, especially the *FOS* gene, which promoted the inflammatory response in the rat myocardium. The processes of glucose to fatty acid transformation and the IL-17 signaling pathway are regulated by transcription factors, especially the transcription factor *HIF1A,* which regulates both processes.

### qRT‒PCR confirmation

**Fig. 8:**
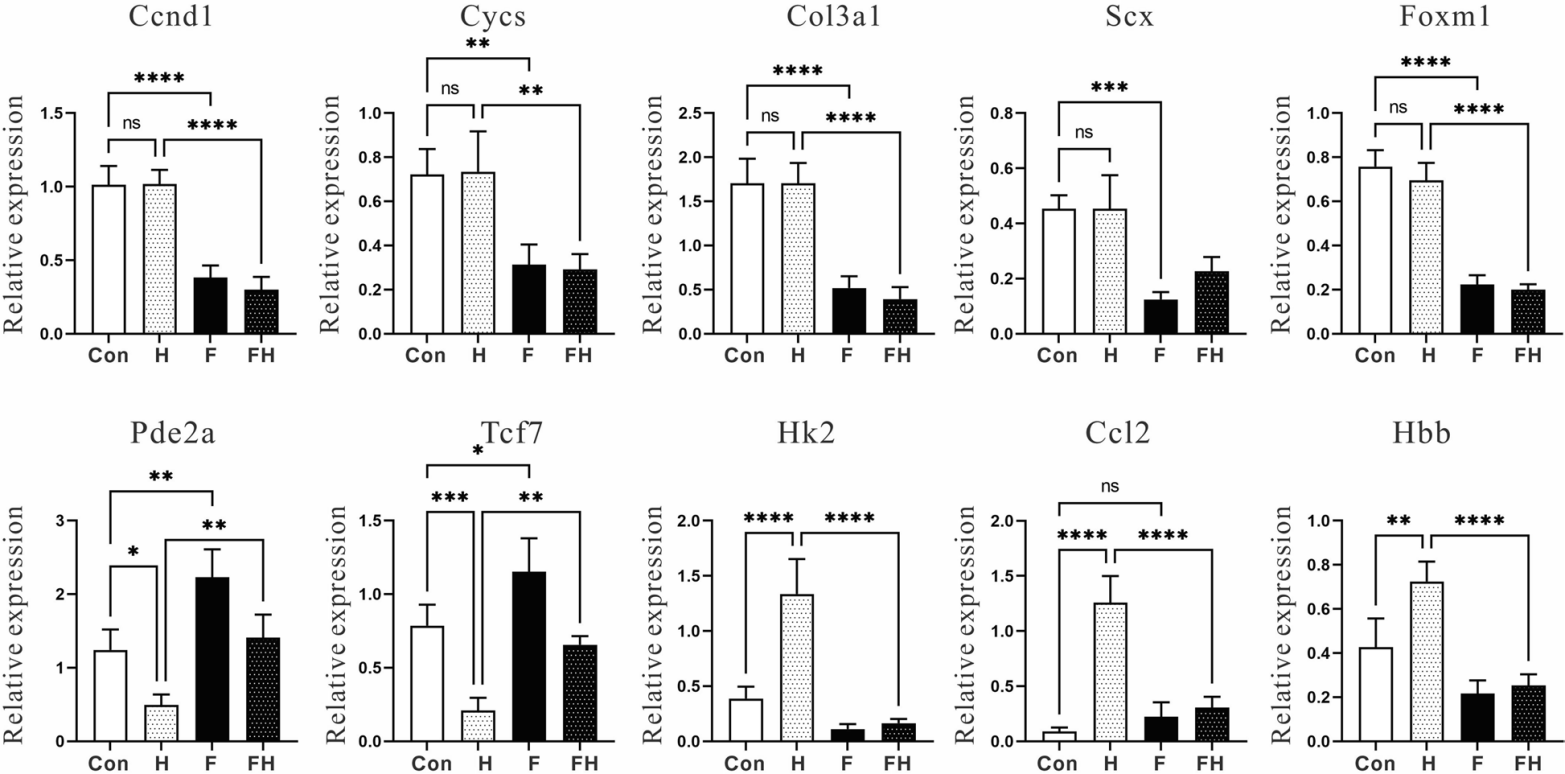
Quantitative real-time PCR (qRT‒PCR) validation of ten DEGs. White and gray bars represent the expression results of RNA-Seq analysis and qRT‒PCR, respectively

To confirm the transcriptome sequencing analysis results, ten related genes were selected for qRT‒PCR validation. For accurate and reliable results, the p53 signaling and collagen pathway-related genes *Ccnd1*, *Cycs*, *Col3a1*, *Scx* and the corresponding transcription factor *Foxm1* (Fig. 5), L-glutamine-related genes and the corresponding transcription factors *Pde2a* and *Tcf7* (Fig. 6), and the carbon metabolism, oxygen, and IL-17 signaling genes *Hk2*, *Hbb*, and *Ccl2* (Fig. 7) were selected. The expression patterns of selected genes determined by qRT‒PCR were consistent with those in the RNA sequencing results, proving the reliability of our sequencing data (Fig. 8).

## Discussion

With the increased demand for entry to extreme HA, methods of rapid adaptation to extreme HA are a challenge but must be explored to protect human health. A previous study proved that fasting could reduce tissue injuries and maintain cardiac function, significantly improving the survival rates of rats at extreme HA [7]. The SD rats undergoing only fasting can survive approximately 10 days at normal temperature (22 °C) and normal attitude, and the survival time decreases to 3 days in cold experiments (2–5 °C) [17]. To investigate the mechanisms of fasting for high-attitude adaptation, we focused on the effect of fasting on the metabolism and gene regulation of hypoxia-sensitive myocardium in rats. Four groups were used to ensure comparable experimental results, and samples were collected from four rats for each group to reduce experimental errors. Consistent with the previous study, the 24 h survival rate of rats at 7620 m was significantly improved from 10 to 85% after fasting pretreatment for 72 h (Table 1). Compared with the rats at normal altitude, the myocardium of rats without pretreatment showed severe damage and was accompanied by a high lethality rate at extreme HA (Fig. 1c), while the impaired features were alleviated with fasting pretreatment, indicating that fasting protects the myocardium cells of rats and thus increases the survival rate of rats at extreme HA.

The detected DMs and DEGs for all two-group comparisons showed consistency. More DMs were identified between the H group and fasting groups (F and FH groups), while the least DMs were identified between the F and FH groups, showing that the effect of extreme HA on rats was reduced after fasting for 72 h. Similarly, the detected DEGs had the same trends as the DMs in these groups, showing the high correlation between metabolism and gene regulation in myocardium cells.

Glycerophospholipids, amino acids, and their metabolites were significantly reduced in the fasting groups, and the most related genes were significantly enriched in multiple mitotic-related pathways, suggesting that fasting attenuated mitotic processes in cardiac tissues and reduced the synthesis of amino acids and lipids (Fig. 5a). Among the related genes, CDK1 plays a key role in the control of the eukaryotic cell cycle and drives cell division [18], while it was significantly downregulated in the fasting groups. In addition, p53 signaling and collagen pathway-related genes were also clustered and had lower expression in the fasting groups. The transcription factors *FOXM1*, *HMGB2*, *E2F2*, and *CENPT* interacted with the identified p53 signaling and collagen pathway-related genes (Fig. 5b). *FOXM1* is expressed in actively dividing cells and is critical for cell cycle progression [19]. *HMGB2* in cardiomyocytes is closely related to cell hypertrophy [20], which may be the reason for the reduction in the synthesis of amino acids and lipids in the fasting groups. Therefore, we hypothesized that fasting caused a substantial reduction in mitotic activity in myocardial tissue, a reduction in the activity of genes related to collagen synthesis, and a substantial reduction in the content of amino acids and lipids, and the reduction in these vital activities was closely related to transcription factors.

L-Glutamine, as a substrate for the synthesis of DNA, ATP, proteins, and lipids, plays a fundamental role in cardiovascular physiology and pathology [21]. L-Glutamine was a particularly important substrate identified in the enrichment results, suggesting that extreme HA could decrease the content of L-glutamine, while fasting could increase its content. In comparison to the H group, L-glutamine-related genes, including TBC1D7, PDE2A, RNF43, and ATP2A3, were upregulated in the other groups,and those genes were enriched in T-cell receptor V(D)J recombination and multiple signal transduction pathways, such as the Hippo signaling pathway, Wnt signaling pathway, cGMP-PKG signaling pathway, and mTOR signaling pathway (Fig. 6). The mTOR signaling pathway is a ubiquitous driver of cell and tissue growth [22]. Previous studies proved that mTOR inhibition can reduce unnecessary ATP consumption, increase ATP reserves, and improve the efficiency of mitochondrial oxygen [7]. *TBC1D7* is involved in the mTOR signaling pathway and can act as an essential suppressor of mTOR signaling [22–24]. It was significantly upregulated in the F group, indicating that fasting could inhibit mTOR signaling by regulating *TBC1D7*. There were also many transcription factors related to L-glutamine, in which TCF7 was also directly involved in T-cell receptor V(D)J recombination, the Wnt signaling pathway, and the Hippo signaling pathway [25–27] (Fig. 6). The upregulation of L-glutamine-related genes and transcription factors in the fasting groups influenced the contents of L-glutamine, with the FH group having the same L-glutamine as that of the Con group. Future studies should focus on the target genes to reveal the mechanisms.

In conclusion, a higher survival rate was proven in SD rats with fasting for 72 h at extreme HA, with lower myocardial damage. In the myocardium, the dynamic contents of DMs between different groups were consistent with the expressions of DEGs, and DM clusters also showed close correlations with DEG clusters. Fasting attenuates mitotic processes in cardiac tissues and reduces the synthesis of amino acids and lipids. Extreme HA decreased the content of L-glutamine in the myocardium, while fasting increased its content even at extreme HA. In addition, abundant fatty acids were detected in rats at extreme HA without fasting pretreatment. For mountain climbers, 24 h fasting or intermittent fasting before climbing may be implemented to protect human health at extreme HA [28]. In addition, the dynamics of metabolism and gene expression under fasting pretreatment and extreme HA provide new insights into the genetic and molecular characteristics of the myocardium, which is critical to developing more potential rapid adaptation methods to extreme HA for climbers in future analyses.

## Data Availability

All data and materials mentioned in this article are available. Sequencing reads have been submitted to the NCBI Sequence Read Archive (SRA) under accession numbers SRR23515896-SRR23515911. The data point of contact is Zhibin Yu.
